# Age-Related Dynamics in Endometrial Vascularity: A Comprehensive Three-Dimensional Ultrasound Evaluation During Follicular and Luteal Phases

**DOI:** 10.3390/jcm14124332

**Published:** 2025-06-18

**Authors:** Badreldeen Ahmed, Justin C. Konje

**Affiliations:** 1 Feto Maternal Centre, Doha P.O. Box 34181, Qatar; profbadreldeen@hotmail.com; 2 Department of Obstetrics and Gynaecology, Qatar University, Doha P.O. Box 2713, Qatar; 3 Department of Obstetrics and Gynecology, Weill Cornell Medicine, Doha P.O. Box 24144, Qatar; 4 Department of Health Sciences, University of Leicester, Leicester LE2 1RQ, UK

**Keywords:** three-dimensional Doppler ultrasound, follicular phase, luteal phase, endometrial vascularity, vascularity flow index, flow index, endometrial volume, endometrial thickness

## Abstract

**Objective:** Transvaginal ultrasonography plays a crucial role in contemporary fertility management, offering insights into uterine and endometrial blood flow. Three-dimensional ultrasonography utilizing power Doppler angiography (3D-CPA) allows precise measurement of endometrial volume and vascular parameters, such as the vascularization index (VI), blood flow index (FI), and vascularization flow index (VFI); variables that indirectly assess endometrial receptivity and integrity. Doppler technology allows for the capture of changes in the uterus induced by hormonal-related fluctuations during the menstrual cycle, revealing a significant correlation between endometrial receptivity and vascularity. Age-related changes in endometrial function are implicated in declining fertility, with limited research exploring this aspect. The aim of this study was to investigate the impact of aging on various ultrasound parameters of the uterus, including endometrial vascularity. **Methods:** A retrospective cross-sectional study of women who attended the Feto-Maternal Centre from January 2022 to December 2023. Each woman whose menstrual cycle was regular underwent 3D ultrasound with power Doppler as part of the routine assessment of the pelvis. Parameters assessed include the VI, FI, and VFI as well as uterine volume, endometrial volume, and endometrial thickness. The women were grouped based on age, and the variables measured in the follicular and luteal phases were compared between the age groups using SPSS version 30 September 2024. **Results:** A total of 907 women (427 follicular and 480 luteal phase) were studied: 297 (131 follicular and 166 luteal) were 20–29 years old; 471 (240 follicular and 231 luteal) were aged 30–39; and 139 (56 follicular and 83 luteal) were aged 40–49. Uterine volume, endometrial volume, and thickness increased significantly and steadily with age. VI, VFI, and FI decreased significantly with age in the follicular phase, but in the luteal phase there was no statistically significant difference in any of these indices with age. **Conclusions:** Uterine volume, endometrial thickness, and endometrial volume increased with age, but the vascular indices decreased with age in the follicular but not in the luteal phase. These age-related changes in endometrial vascularity may partly explain the decrease in age-related fertility. Further research is needed to comprehensively explore the complexities of uterine aging and its implications for female fertility.

## 1. Introduction

Transvaginal ultrasonography is vital in modern fertility management, enabling the monitoring of uterine and ovarian physiological changes, including follicular development, endometrial development, and blood flow during assisted reproduction techniques (ART). Three-dimensional ultrasonography using power Doppler angiography (3D-CPA) allows for the accurate measurement of endometrial volume and vascular parameters, including the vascularization index (VI) (indicating the number of blood vessels), the blood flow index (FI) (reflecting blood flow strength over time), and the vascularization flow index (VFI) (indicating the combined blood flow and vascularization in the region). Together, these parameters indirectly reflect endometrial blood supply [1].

Doppler technology can demonstrate fluctuations in blood supply that are related to hormonal changes during the menstrual cycle. This is performed by means of color Doppler imaging. Previous reports noted a significant correlation between endometrial receptivity and endometrial vascularity and suggest that such observations help in the understanding of the factors that may aid embryonic growth and development [2]. A study by Choi et al. showed that inadequate blood flow causes endometrial and sub-endometrial hypoxia, thus lowering receptivity and reducing successful implantation as well as increasing spontaneous miscarriages [3].

Modern lifestyles have meant that an increasing number of women delay having children. Increasing age at conception is thus now a critical risk factor for female infertility. While maternal age is widely recognized as a key factor in declining oocyte quality and an increased incidence of chromosomal abnormalities in both oocytes and embryos, all of which are associated with declining fertility, age-related changes in endometrial physiology and function may also significantly affect implantation and successful pregnancy rates and overall female fertility [4]. Research on endometrial physiology and function as it pertains to implantation and successful pregnancies is, however, limited. Investigating endometrial physiology and function could enhance our comprehension of endometrial aging from a biological and clinical perspective and perhaps pave the way for not only modifying these physiological changes to improve implantation and successful pregnancies but also allowing for the possibility of investigating interventions that could reduce the risks associated with age-related female infertility. Doppler imaging allows a non-invasive means of studying endometrial vascular changes, which can be considered a proxy to physiology and function. Previously we investigated endometrial vascularity in normally menstruating women and those in other physiological states using the same technology [5]. The aim of this study was to investigate the effects of aging on the different ultrasound (US) parameters that we used in our previous study to assess endometrial vascularity, building on our findings and expertise [5].

## 2. Methods

We previously published our methods in a cross-section study [5], which will be briefly outlined here. Since this was a retrospective study, the women were not required to give consent as their data were accessed anonymously. Approval for this study was obtained from the Feto Maternal Centre Institutional Review Board (IRB Ref. No. of FMC/IRB/003/23rd March 2023).

### 2.1. Experimental Design

This was a cross-sectional retrospective study of patients seen at our center between 2022 and 2023. All patients attending gynecological consultations routinely have a transvaginal 2D/3D ultrasound scan for the assessment of the pelvis. At this procedure the endometrial vascularity is also obtained with power Doppler. All the imaging was performed by experienced clinicians. For the purposes of this study, we opted to review only the images from two (BA and JCK) of the consultants to minimize inter-observer variability.

The records of those who met the inclusion criteria were retrieved, and various variables were extracted. The inclusion criteria were (a) healthy women attending for gynecological check-ups, typically prior to embarking on pregnancy (74% of the cohort) or undergoing ART (26% of the cohort); (b) between the ages of 20 and 50 years and having normal menstrual cycles; (c) not on any hormonal treatment for at least the previous 3 months; and (d) certain of their last menstrual period. The imaging performed by the consultants BA and JCK was with a General Electric (GE) Voluson 10 (Illinois, USA) ultrasound machine using a 5–9 mHz 3D transvaginal probe. The wall filter was set at low and the pulse repetitive frequency (PRF) at 0.7. A longitudinal view of the uterus was obtained in 2D. The 3D program was then switched on, and an image was obtained with the power Doppler mode on. A multiplanar image was generated and stored for later analysis [5].

### 2.2. Data Acquisition

Firstly, a truncated sector defining the area of interest was obtained with the volume mode on. The probe was next moved and adjusted, and the sweep angle set to 90° so that a complete uterine volume encompassing the entire sub-endometrium was obtained [5,6]. The women were advised to remain as still as possible to minimize inappropriate movements of the transducer that would generate noise in the image during this period [5]. A three-dimensional dataset was next acquired using the medium speed sweep mode. The resulting multiplanar display was examined to establish that the area of interest had been captured completely, with special attention paid to the coronal image in the C plane, specific to the 3D ultrasound, which provides more spatial information than the transverse or longitudinal planes [5]. If the volume was complete with no artifacts, the images were then stored on the hard drive of the ultrasound machine. For cases where images were judged to have failed pre-defined set criteria (e.g., the presence of artifacts), imaging was repeated until satisfactory ones were obtained.

### 2.3. Data Analysis

A single operator (a technician within our team) retrieved and analyzed the stored 3D images to generate the various variables studied. We used the Virtual Organ Computer-Aided Analysis (VOCAL^TM^) (manufactured by GE Illinois, USA) program for the 3D endometrial volumetric analysis. This program allows the manual use of a standard computer mouse to define the volume of interest as the dataset is rotated about a central axis [6]. Plane C (coronal image) was used for all the measurements, which were made manually. This plane was obtained by rotating plane A (longitudinal plane) using the 9° rotation step. Because the dataset is rotated by 180°, the 9° rotation makes available 20 planes to calculate individual volumes. This has been shown to represent the best compromise between reliability, validity, and time to define initial volume [7].

After defining the endometrium, the power Doppler signal within it was quantified through the ‘histogram facility’ of the program, which employs specific mathematical algorithms to produce three indices of vascularity [8]. which are representative of either the percentage of power Doppler data within the defined volume (the VI; vascularization index), the signal intensity of the power Doppler information (the FI; flow index), or a combination of both factors (the VFI; vascularization flow index) and have been suggested as representative of vascularity and flow intensity [9]. Table 1 shows the definitions of these indices. Following assessment of the endometrium, the sub-endometrium was next examined by using `shell imaging’, which allows for the generation of a variable contour by the user that parallels the originally defined surface contour. We used shell imaging in this study to define a 3D region within 5 mm of the originally defined myometrial/endometrial contour, and then the power Doppler signal within this sub-endometrial region was quantified. Although this is an arbitrary distance, it is one that reflects the inner third of the myometrium and the region supplied by the radial arteries [10].

### 2.4. Statistical Analysis

Statistical analysis was performed by using SPSS 29.0.0.0. The data were de-identified before analysis. Patients were divided into 3 groups based on age. Group 1 (20–29 years), group 2 (30–29 years), and group 3 (40–49 years). Outliers, defined as those with values > 2 standard deviations from the mean, were excluded from this analysis. The one-way ANOVA test was used to test for differences in the 6 main parameters: uterine volume, VI, FI, VFI, endometrial volume, and endometrial thickness between the age groups within each phase of the menstrual cycle. Student’s *t*-test was used to test differences in the 6 parameters between the follicular and luteal groups. To compare the measured variables between the follicular and luteal phase groups, we used the Games–Howell post hoc test. This is a post hoc analysis method for undertaking multiple comparisons involving two or more samples. We used it because it does not assume equal sample sizes and variances among the studied groups. It is used to identify which groups are significantly different if the ANOVA test is significant. Results are presented as mean and standard deviation where data were normally distributed. The Kolmogorov–Smirnov test was used to test for normality of data. A *p*-value of <0.05 was considered statistically significant.

## 3. Results

A total of 907 women were included in this study—427 (47.1%) in the follicular phase and 480 (52.9%) in the luteal phase. There were 297 (32.7%) (131 follicular and 166 luteal) in the age group 20–29 years, 471 (51.9%) (240 follicular and 231 luteal) in the age group 30–39, and 139 (15.3%) (56 follicular and 83 luteal) in the age group 40–49. The variables studied were next analyzed based on age and the phase of the menstrual cycle. The number of outliers removed was 12 at 20–29 years, 9 at 30–39 years, and 11 at 40–49 years. As these numbers are relatively small, they are unlikely to have affected the overall analyses.

### 3.1. Follicular Phase

Table 2 shows the mean parameters measured in the 427 women studied in the follicular phase. A one-way ANOVA was used to compare the means between the three different age groups. Age had a significant negative effect on the variables measured. The effect size was moderate for vascularization index (η^2^ = 0.034) and vascularization flow index (η^2^ = 0.023) and mild for flow index (η^2^ = 0.017). Age also had a significant effect on endometrial thickness, volume, and uterine volume. The effect size was moderate for endometrial thickness and volume (η^2^ = 0.042 and 0.073) and strong for uterine volume (η^2^ = 0.235).

### 3.2. Luteal Phase

Table 3 shows the mean parameters measured in the 480 women in the luteal phase. A one-way ANOVA was used to compare the means between the three different age groups. Age had no significant effect on the vascular indices (VI, VFI, and FI) but had a significantly positive effect on endometrial thickness, volume, and uterine volume. The effect size was very mild for endometrial thickness and volume (η^2^ = 0.01 and 0.02, respectively) and moderate for uterine volume (η^2^ = 0.12).

### 3.3. Comparison of Follicular vs. Luteal

**Figure 1:**
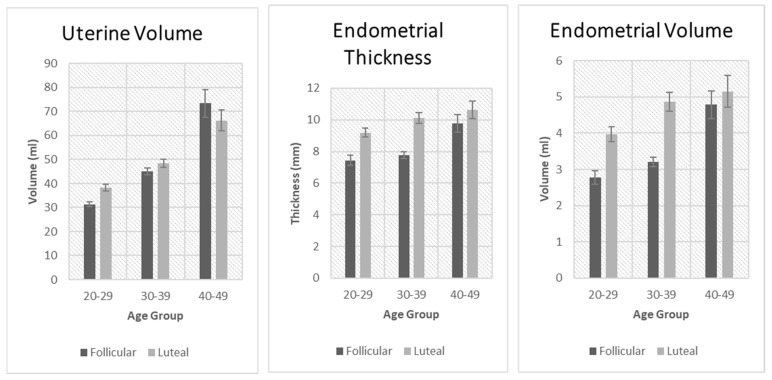
Comparison of uterine volume, endometrial thickness, and endometrial volume between the follicular and luteal phase groups.

**Figure 2:**
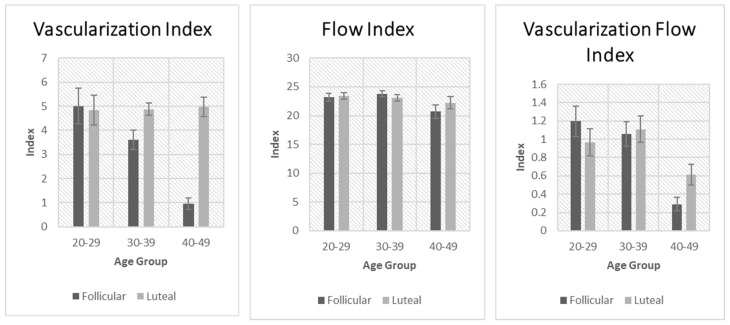
Comparison of vascularization index, flow index, and vascularization flow index between the follicular and luteal phase groups.

These comparisons are shown in Figure 1 and Figure 2. Uterine volume was significantly affected by age in both the luteal and follicular phases. There was a significant increase in uterine volume with age. Vascularization index was significantly decreased with age but only in the follicular phase. The same applied to the flow index and vascularization flow index. Endometrial thickness was significantly affected by age in the follicular and the luteal phases. Endometrial volume was also significantly affected by age in the follicular and the luteal phase.

## 4. Discussion

The main findings in this study were that age had a significant effect on all the variables (VF, FV, VFI, endometrial thickness, volume, and uterine volume) studied in the follicular phase. While all the vascular indices decreased with age, the measured dimensions (endometrial thickness and volume and uterine volume) increased with age. In the luteal phase, the vascular indices were unaffected by age, but the measured dimensions increased with age. It is possible that the changes in measured uterine dimensions reflect repeated stimulation by estrogens over the course of the women’s reproductive life. Estrogen stimulates myometrial hypertrophy, which would account for the increased uterine volume. By limiting our study population to strictly those who had regular cycles, were neither over nor underweight, and had no endocrine or medical disorder, we reduced the number of possible confounding factors, especially those that might increase peripheral estrogen (for example, in obese, diabetics, and hypertensive individuals), which in turn may affect endometrial vascularity.

Uterine aging has not been very well studied, although described changes have been associated with abnormal hormonal levels that occur especially around menopause [11]. Furthermore, endometrial aging has been shown to negatively impact implantation, clinical pregnancy, and live birth rates. Our data show that age is associated with changes not only in endometrial morphology (as measured by ultrasound) but also in its vascularity. Endometrial vascularity emerges as a crucial factor in successful implantation [12]. In our data, vascularity diminished with age, notably declining after 39 years, coinciding with a significant drop in implantation rates. In fact, the highest vascularity was observed in the 20–29 age group, which is in general regarded as the age of optimum reproductive outcome [13]. Recently, there have been some studies on the significance of age-related epigenetic alterations using the epigenetic clock to predict the biological age of the endometrium in certain endometrial disorders and in infertility [4]. A combination of these and vascularity changes could have a compounding effect on reducing successful implantation rates with aging.

The most notable morphological change in the aging uterus is uterine collagen deposition. This is associated with chronic inflammation involving various mediators like interleukins, growth factors, oxidative stress products, and senescent cells. Estrogen plays a role in fibrosis and senescence activation through specific signaling pathways. Senescence is observed in the aging uterus, resulting in cells with reduced proliferation. Studies in mice and humans have also shown that aging is associated with changes in gene expression related to cell proliferation and the appearance of senescent cells [11]. Studies that have investigated the impact of age on endometrium using functional genomics have suggested dysregulation of key genes and processes required for endometrial competence occurring with age [14]. These dysregulated genes found in women older than 35 years included those involved in endometrial receptivity acquisition, cell cycle arrest, maintenance of telomeric length, and genomic stability [14]. Other studies have shown age-related decline in factors such as those involved in decidualization and stromal cell proliferation, as well as an increase in epigenetic molecular age with chronological age [14].

Our utilization of 3D Doppler ultrasound to elucidate the changes in the aging endometrium was motivated by several factors. In recent years, there has been significant progress in the development of 3D color Doppler ultrasound technology, marked by enhanced resolution and expanded capabilities for measuring parameters previously beyond reach. Furthermore, transvaginal ultrasound is unique as a non-invasive diagnostic modality. Lastly, the simplicity and cost-effectiveness of ultrasound measurements facilitate widespread applicability and generalizability.

Our findings of a significant increase in uterine volume with aging are consistent with studies that have shown a peak in uterine volume at around 35–40 years, which then declines by 50–60 years [15]. Uterine growth generally continues during the reproductive years and ceases at menopause, ultimately regressing in size to approximate its pubertal form. This direct correlation between age and uterine size/volume was observed in numerous other studies and may be attributed to the decrease in ovarian estrogen secretion that occurs with aging. Studies have also shown a positive correlation between parity and uterine volume, which could also be an underlying factor that plays a role in increasing uterine volume [16]. These findings were summarized in the systematic review and meta-analysis by Marti-Garcia et al. [14].

Endometrial volume was significantly affected by age in both the follicular and the luteal phases, as seen in Table 2 and Table 3. Endometrial volume, previously not well studied as a proxy for assessing endometrial receptivity, is increasingly being considered as a comprehensive marker of receptivity. Recent ultrasound advances have enabled research on its correlation with embryo implantation and indeed considering it a valuable marker for evaluating endometrial receptivity. Endometrial volume has been suggested as a potential predictor for successful in vitro fertilization (IVF), with a study using a 3.2 mL cut-off on the day of embryo transfer reporting 80% sensitivity and 77.1% specificity [17]. Another study suggested endometrial volume as a promising alternative to using traditional thickness in predicting IVF success, revealing significant differences between pregnant and non-pregnant women on crucial treatment days [18]. However, Boza et al. [19], in their study of 142 patients, contradicted this by showing that 3D transvaginal ultrasound-assessed endometrial volume was not a reliable predictor in single blastocyst embryo transfer cycles, with an AUC of 0.48. This discrepancy underscores the need for further research to establish a consensus on the efficacy of endometrial volume as a predictive marker in assisted reproductive technologies (ART). In summary, while endometrial volume was unlikely to serve as a predictive factor for pregnancy, it is worth noting that patients with an endometrial volume less than 2.0–2.5 mL may experience a significantly reduced pregnancy rate [20].

Endometrial thickness was also significantly affected by age in both the follicular and the luteal phases, as shown in Table 1 and Table 2. This increase in endometrial thickness with aging has also been observed in previous studies. One study noted that women aged 32–36 years and 37–45 years had a maximum thickness of 15.3 mm and 15.9 mm during the secretory phase, respectively, surpassing those of younger age groups (21–25 and 26–31 years) with a maximum thickness of 12.1 mm and 13.4 mm, respectively. However, the determination of the impact of age-related variations in endometrial thickness on pregnancy rates has not been conclusively established [21,22].

Our findings of changing endometrial vascularity with age and the phase of the menstrual cycle are consistent with other studies that used other methods for assessing endometrial vascularity. In a systematic review of 17 studies, it was found that 7 reported decreased blood flow supply and poor morphology with increasing age [14]. Indeed, in two of the studies, there was significant mineral (calcium, sodium, and phosphorus) accumulation in the uterine arteries of older women [23,24]. with other studies reporting on altered morphology and structure of the uterine vasculature with age—a higher intimal area and lower medial area of the uterine arteries [25,26,27,28]. Amin et al. [29] found that the uterine veins dilated until age 41–50 years and thereafter constricted. Furthermore, some endometrial functional studies showed reduced vascular and flow indices with increasing age [25,27,28]; however, two others failed to demonstrate any changes. In studies using Doppler of the uterine arteries, no changes in resistance and/or pulsatility indices were noted with relation to age [30,31,32]; however, two showed an increase in pulsatility or resistance indices.

We observed statistically significant associations with aging for flow index, vascularization index, and vascularization flow index (indices that have been shown in studies to be a useful parameter in the prediction of pregnancy in frozen embryo transfer cycles [33]), specifically in the follicular phase (as opposed to the luteal phase). This distinction was an intriguing and surprising observation. What could be responsible for this? A study by Burger et al. [34] showed that estradiol (E2) levels in the follicular phase were elevated in older women (>45 years) compared to younger women (20–35 years) [33]. This was mainly explained through a hypothesis whereby the feedback mechanism controlled by inhibin B was affected earlier in the climacteric as compared to E2 secretion. With aging, as follicle numbers decrease, inhibin levels decrease, leading to increased FSH secretion. Over time, this will drive an increase in E2 levels and thus enhance endometrial growth. The close correlation between E2 levels and endometrial proliferation suggests that abnormal E2 concentrations in older women may have a detrimental effect on endometrial growth and thickness. Moreover, during the secretory phase, serum levels of E2 and P4 decrease progressively with age [35], which can further influence cellular differentiation during endometrial receptivity and neovascularization. In fact, the effect size of age on vascularization index was moderate but only mild for flow, suggesting that age is likely to have a greater effect on vascularization than on flow. The age-dependent variation in E2 and P4 levels may also have a considerable impact on the endometrium by altering the endometrial expression of their corresponding cellular receptors. Furthermore, age-related endometrial downregulation of ER and P4 receptors (PR) has been demonstrated in the endometrial tissue. Additionally, the decreased capacity of the endometrium to take up these steroids [36,37,38] was associated with increased collagen and fibrosis in endometrial tissue [39,40]. These changes could possibly explain the discrepancy observed in the three ultrasound parameters (FI, VI, and VFI) between the follicular and luteal phases of these women [22].

What would be the implications of the changing endometrial vascularity (which has been suggested by some as a proxy for endometrial receptivity) on natural conception or ART pregnancy rates? Age-related decline in female fertility is reported to clinically start from the age of 35–40 years [41], a period when the vascularity indices also fall significantly. This fall in female fecundity with increasing age has traditionally been attributed to ovarian dysfunction (decrease in ovarian reserve associated with poor quality oocytes), which results in embryos (from natural conception or ART cycles) that in a high proportion of cases are either non-implantable or are chromosomally abnormal [42]. Interestingly, this age-related decline in fertility is not always secondary to ovarian factors, as currently available ART-related advances, such as oocyte donation and competent embryo selection by screening by preimplantation genetic testing for chromosomal abnormalities, have not necessarily resulted in a significant increase in pregnancy rates after ART in older women [4]. This is because the aging endometrium with its decreasing vascularity, as shown from our findings and other studies, is likely to have a significant negative impact on implantation, clinical pregnancy, and live birth rates in women of advanced age. This being the case, we believe that focusing on oocyte donation protocols alone and ignoring those that improve receptivity might not be as effective in significantly increasing pregnancy rates in older women.

Finally, there are factors other than endocrine that could possibly explain our findings. It is well recognized that in the aging endometrium, alterations occur at various levels, including molecular, cellular, and histological levels, that negatively affect the endometrium and its receptivity. Studies using polymerase chain reaction (PCR) or Western blot to investigate molecular changes in the endometrium with age have shown that levels of MK167 (a molecular marker of proliferation Ki67) and MCM2 (Minichromosome Maintenance Complex Component 2) were reduced in older women, implying alteration in cell cycle proliferation with age [43]. Furthermore, Maia-Filho et al. [44] found downregulation of E-cadherin, suggesting that the adhesion process is altered with age, but the expression of other adhesion markers such as MUCIN 1 (Mucin 1), leukemia inhibitory factor (LIF), integrin B3, and glycoconjugate sugar residue content and distribution (related to peri-implantation feto-maternal molecular recognition) did not change. Interestingly, levels of HOXA10 (Homeobox A10), a well-known marker of endometrial receptivity, were lower in older women, while levels of endometrial apoptosis and senescence were elevated, and the proportion of p16-positive senescent cells in uterine epithelium and endometrial glands was higher [45,46,47]. Additionally, advanced age influences cellular senescence, which plays an important role during the initial phase of implantation.

Aging is a complex and multifactorial biological process that promotes time-dependent deterioration of tissue function across multiple organ systems. It is a result of cumulative changes caused by several stimuli, such as telomere shortening, epigenetic changes, DNA damage, oxidative stress, chronic mitogen signaling, and mitochondrial dysfunction over a period, which leads to cell proliferation arrest and a decline in cellular function [48,49,50]. At the level of a single cell, aging is closely associated with the irreversible underlying biological process called cellular senescence [51,52,53]. Additionally, as a response, the senescent cells with their damaged DNA secrete several growth factors, chemokines, pro-inflammatory cytokines, and matrix metalloproteinases, which are collectively termed the ‘senescence-associated secretory phenotype (SASP)’ [49,53,54] that may cause an aberrant pro-inflammatory response known as ‘inflammaging’. These changes ultimately lead to a thickened endometrium with reduced vascularity and the larger uterus measured in ours and other studies with advanced age. Such changes lead to endometrial dysfunction [55,56] and the ensuing reduced receptivity. 

## 5. Strengths and Limitations

The main strength of our study is the large number of patients involved and the restriction of the study population to those without any possible confounding variables. Furthermore, limiting the scanning to two clinicians minimized inter-observer variability.

There are several limitations to our study. By its very nature, a retrospective study is more likely to be associated with selection bias, missing data, and a lack of appropriate controls. It would have been ideal to have a population of women with other confounders that are well recognized to influence endocrine biology, such as obesity, hypertension, and medical disorders such as diabetes. Additionally, since parity is well known to influence changes in uterine size, not including this variable in our analysis could limit the interpretation of our conclusions. This factor is being taken into consideration in our prospective studies. Variability among patients, both in medical history and demographics, may confound this study’s outcomes. The stringent inclusion and exclusion criteria, focusing solely on women undergoing routine ultrasound at one center, may limit the external validity of our findings. By removing outliers, we excluded data from a few of our study population. While this is an acceptable statistical approach to data analysis, it must be recognized that such data may indeed be real and might have significantly skewed the overall spread of the data. The limitations in this study would be addressed by prospective longitudinal studies that focus on various cohorts of women, such as those attending with infertility and those attending for routine assessment prior to infertility. Control groups would be identified as women who had achieved pregnancy spontaneously and matched for age, parity (which is likely to have an effect on uterine size), weight, and other variables to compare the variables between the groups. It would be interesting to not only determine the various parameters we have measured but also to relate them to success rates at ART—i.e., assess the uterine variables prior to starting superovulation cycles.

## 6. Conclusions

Our findings of decreasing vascularity but increasing uterine morphometry with advancing age up to menopause suggest that there are likely to be more than hormonal factors involved in influencing endometrial biology. The findings demonstrate a correlation between uterine aging and vascular and morphological changes and provide a possible explanation for diminished fertility with age. While age is an important factor in the quality of eggs produced, its impact on the endometrium, as shown in ours and other studies, should be considered as a factor that decreases fertility with age. This is important in counseling women of advanced age as donor eggs, while they may overcome the quality of the eggs, do not bypass that of the aging endometrium. A combination of factors is involved in senescence—changes that occur with age. The cumulative interaction of these factors results in the various changes we reported. We believe that these contribute to the decrease in fertility with age and emphasize the need to investigate approaches to improving endometrial vascularity and senolysis to retain or improve endometrial receptivity. Insights into age-related alterations in endometrial vascularity, thickness, and volume enhance our understanding of factors impacting reproductive outcomes. However, this study’s limitations, such as its retrospective nature and clinic-specific focus, necessitate cautious interpretation. Further research is imperative to thoroughly investigate the intricacies of uterine aging and its implications for female fertility.

## Figures and Tables

**Table 1:** Definition of power Doppler quantification indices.

Vascularity Index	Denotation	Description
Vascularization flow index	VFI	Weighted color values/(color values + greyscale values)
Vascularization index	VI	Color values/(color values + greyscale values)
Flow index	FI	Weighted color values/color values

**Table 2:** Summary of ultrasound-measured parameters in the follicular phase with mean and SD by age group and one-way ANOVA, *p*-values with F-values.

Follicular Phase								
US Parameter	20–29		30–39		40–49		F Value	*p*-Value
	M	SD	M	SD	M	SD		
VI	5.0	8.4	3.6	6.1	0.9	1.8	7.3	<0.001 *
VFI	1.2	1.9	1.1	2.0	0.3	0.6	4.8	0.009 *
FI	23.2	7.7	23.8	7.5	20.7	8.7	3.6	0.028 *
EMT	7.4	3.5	7.8	3.3	9.8	4.2	9.3	<0.001 *
EMV	2.8	2.1	3.2	2.1	4.8	2.8	16.5	<0.001 **
UV	31.4	11.8	45.0	21.1	73.5	42.6	64.6	<0.001 **

VI = vascularization index; VFI = vascularization flow index; FI = flow index; EMT = endometrial thickness; EMV = endometrial volume; UV = uterine volume; US = ultrasound. * significant *p*-values (*p* < 0.05), calculated using the one-way ANOVA test. ** highly significant *p*-values (*p* < 0.001), calculated using the one-way ANOVA test.

**Table 3:** Summary of ultrasound parameters in the luteal phase with means and standard deviations by age group and one-way ANOVA *p*-values with their F-values.

Luteal Phase								
US Parameter	20–29		30–39		40–49		F Value	*p*-Value
	M	SD	M	SD	M	SD		
VI	4.8	8.0	4.9	3.9	5.0	3.6	0.0	0.986
VFI	1.0	1.8	1.1	2.2	0.6	1.0	2.0	0.137
FI	23.4	7.6	23.1	8.5	22.2	9.6	0.6	0.576
EMT	9.2	3.7	10.1	5.0	10.6	5.0	3.2	0.042 *
EMV	4.0	2.6	4.9	3.9	5.2	3.9	4.3	0.015 *
UV	38.3	16.8	48.4	24.3	66.3	39.0	33.3	<0.001 **

VI = vascularization index; VFI = vascularization flow index; FI = flow index; EMT = endometrial thickness; EMV = endometrial volume; UV = uterine volume; US = ultrasound. * significant *p*-values (*p* < 0.05), calculated using the one-way ANOVA test. ** highly significant *p*-values (*p* < 0.001), calculated using the one-way ANOVA test.

## Data Availability

The original contributions presented in this study are included in the article. Further inquiries can be directed to the corresponding author(s).

## Synopsis

Endometrial vascularity decreases with age in the follicular phase but is unchanged in the luteal phase. Endometrial volume, thickness, and uterine volumes increase with age.
